# An Amylase-Like Protein, AmyD, Is the Major Negative Regulator for α-Glucan Synthesis in *Aspergillus nidulans* during the Asexual Life Cycle

**DOI:** 10.3390/ijms18040695

**Published:** 2017-03-27

**Authors:** Xiaoxiao He, Shengnan Li, Susan Kaminskyj

**Affiliations:** 1 Key Laboratory of Molecular Epigenetics, Ministry of Education, Institute of Genetics and Cytology, Northeast Normal University, Changchun 130024, Jilin, China; 2 Jilin Institute of Biology, Changchun 130012, Jilin, China; lishengnan8435@hotmail.com; 3 Department of Biology, University of Saskatchewan, 112 Science Place, Saskatoon, SK S7N 5E2, Canada; susan.kaminskyj@usask.ca

**Keywords:** AmyD, α-glucan, *A. nidulans*, cell wall, fungal virulence

## Abstract

α-Glucan affects fungal cell–cell interactions and is important for the virulence of pathogenic fungi. Interfering with production of α-glucan could help to prevent fungal infection. In our previous study, we reported that an amylase-like protein, AmyD, could repress α-glucan accumulation in *Aspergillus nidulans*. However, the underlying molecular mechanism was not clear. Here, we examined the localization of AmyD and found it was a membrane-associated protein. We studied AmyD function in α-glucan degradation, as well as with other predicted amylase-like proteins and three annotated α-glucanases. AmyC and AmyE share a substantial sequence identity with AmyD, however, neither affects α-glucan synthesis. In contrast, AgnB and MutA (but not AgnE) are functional α-glucanases that also repress α-glucan accumulation. Nevertheless, the functions of AmyD and these glucanases were independent from each other. The dynamics of α-glucan accumulation showed different patterns between the AmyD overexpression strain and the α-glucanase overexpression strains, suggesting AmyD may not be involved in the α-glucan degradation process. These results suggest the function of AmyD is to directly suppress α-glucan synthesis, but not to facilitate its degradation.

## 1. Introduction

α-1,3-Glucan (hereafter, α-glucan) and β-1,3/1,6-glucan (hereafter, β-glucan) are major cell wall components for many filamentous fungi, as well as for many yeasts [1]. However, they have very different impacts on fungal cells. β-glucan is essential for fungal cell survival, at least for species of *Aspergillus* and *Candida* [2,3], hence, β-glucan synthase inhibitors (echinocandins) are used clinically to treat systemic aspergillosis and candidiasis [4]. In contrast, α-glucan has been found to be important for the morphology of *Schizosaccharomyces pombe* [5], particularly for cell integrity at cytokinesis [6]. For other fungal species, α-glucan synthase deleted strains cause minor or no phenotypic change [7,8,9,10]. Nevertheless, accumulated evidence has suggested α-glucan has a role in host‑pathogen interaction [11,12,13], which is important for successful pathogenesis. Thus, treatments that could eliminate fungal α-glucan might be able to prevent fungal infection. Unfortunately, to our knowledge, no drug has yet been developed that targets α-glucan synthase. As an alternative strategy to blocking the synthesis of α-glucan, we could potentially degrade α-glucan from fungal cell walls to achieve the same purpose.

α-1,3-Glucanase (hereafter, α-glucanase) expressed by fungal cells can recycle α-glucan from their cell walls. In *S. pombe*, α-glucanase (*Sp*Agn1p) was shown to have an endo-catalytic hydrolysis ability on α-glucan (hydrolyzing α-glucan into pentasaccharides), and was thereby important for successful cell division [14]. *Paracoccidioides brasiliensis* α-glucanase (*Pb*Agn1p) had comparable activity [15]. In *Trichoderma* species, two α-glucanases have been characterized, and these were suggested to have antifungal effects because their expression was highly induced under antagonistic conditions [16,17]. Together this is strong evidence that α-glucanase has the potential to eliminate α-glucan from fungal cell walls. Consistent with this, a strain of transgenic rice plants that expressed a bacterial α-glucanase was more resistant to *Magnaporthe oryzae* [12]. Therefore, degrading α-glucan from fungal cell walls is a possible way to prevent fungal infection, and characterization of glucanase-like proteins will provide additional useful information about this major but enigmatic wall carbohydrate.

In our previous study, we reported that an amylase-like protein (AmyD) repressed α-glucan synthesis in *A. nidulans* [10]. Amylase-like proteins with similar effects were also seen in *S. pombe* [18] and *A. niger* [19]. These data suggest some amylase-like proteins may have the same potential as α-glucanase to eliminate α-glucan from fungal cell walls. To explore this, we studied the function of AmyD along with two other amylase‑like proteins and three α-glucanases in the α-glucan degradation process. We found that AmyD localized at the cell membrane and was the only amylase-like protein in *A. nidulans* that could repress α-glucan accumulation. In addition, AmyD function was independent from that of α-glucanase. All functional α-glucanases maintained low expression levels in the *A. nidulans* asexual life cycle. Thus, AmyD is the major negative regulator for α-glucan accumulation in *A. nidulans* during the asexual life cycle, and may have a potential to prevent fungal infection.

## 2. Results

### 2.1. AmyD Mainly Localizes at the Cell Membrane

**Figure 1:**
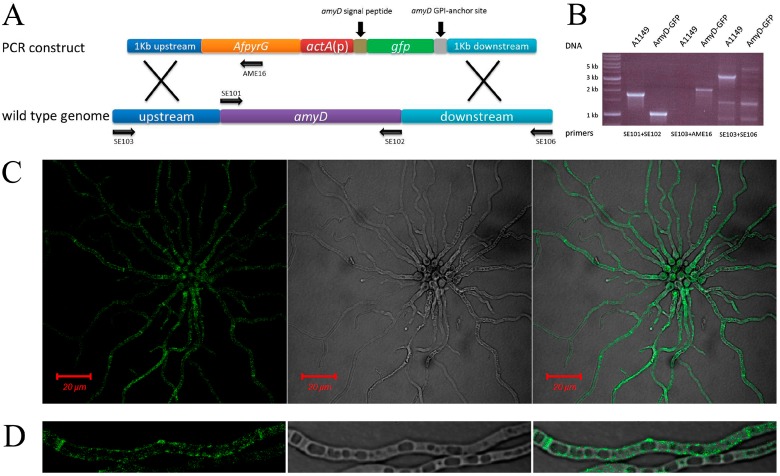
Localization of AmyD associates with cell membrane; (**A**) Strategy of chimera-AmyD-GFP strain construction. The amylase-like domain of AmyD was replaced by a GFP, whereas the localization determining elements of AmyD were maintained. The localization of AmyD corresponded to a chimera-GFP signal; (**B**) PCR verification of AmyD-GFP strain. Primers targeting sites were labeled in (**A**). PCR using SE101 + SE102 primers confirmed the replacement of *amyD* by *gfp*. PCR using SE103 + AME16 and SE103 + SE106 primers confirmed the integration of the construct at the designed place. No ectopic insertion was found in the AmyD-GFP genome; (**C**) the GFP signal was examined using a Zeiss META501 confocal epifluorescence microscope at 63 × NA 1.2 or 25 × N.A. 1.0 objective lens. Confocal imaging used 488 nm excitation with emission controlled by a BP 505–530 nm filter; and (**D**) a magnified hypha image showed the GFP signal strongly associated with septa and the cell membrane.

In our previous work, we reported that AmyD repressed α-glucan synthesis, and so we hypothesized that AmyD might work together with one or more α-1,3-glucanases to degrade α-glucan [10]. In order to understand the mechanism of AmyD as an α glucanase, it is important to determine its localization. However, due to hindrance from the AmyD protein and the GPI-anchor site, we did not get a visible signal when the green fluorescent protein (GFP) was tagged after the signal peptide (between the 26th and 27th amino acids) or after the GPI-anchor site. To solve these problems, we replaced the amylase domain of AmyD (from the 63rd to the 507th amino acid) by a GFP (for details of the strain construction and verification, see Figure 1A,B). The N-terminal signal peptide and C-terminal GPI-anchor site of AmyD were maintained, which were the elements that determined AmyD localization. The same strategy has been reported to solve the localization issue of other GPI-anchored proteins [20,21]. The GFP signal for this strain (chimera-AmyD-GFP) showed strong association with septa (Figure 1C) and patchy localization with the cell membrane (Figure 1D), which was consistent with the localization of a GPI-anchor protein. Therefore, AmyD may be involved in α-glucan degradation based on its plasma-membrane localization.

### 2.2. AmyC and AmyE Do Not Affect α-Glucan Accumulation

In the *A. nidulans* genome, there are two putative/annotated amylase-like proteins, AmyC (encoded by ANID4507) and AmyE (encoded by ANID6324) that share high sequence similarity with AmyD. Like AmyD, both are predicted to have an N-terminal signal peptide and C‑terminal GPI‑anchor site [22]. Their overall amino acid sequence identities to AmyD are 59% (AmyC) and 45% (AmyE), respectively (Figure S1), suggesting they could have conserved functions with AmyD.

To study their functions, we first examined their expression levels to see when we could expect to detect their activities. Samples were grown in shaken liquid medium and in static liquid medium for 14 h and 24 h respectively. In shaken liquid medium, *A. nidulans* grows vegetatively (hyphal elongation only) but does not undergo colony development. In static liquid medium, *A. nidulans* undergoes its complete asexual life cycle. Conidiophores with conidia were seen when we collected the static samples at 24 h. Unlike *amyD* that had a general high basal expression [10], we found *amyC* and *amyE* maintained low expression levels throughout the *A. nidulans* asexual life cycle (Table 1). This conclusion was based on high Ct values of *amyC* and *amyE* in qPCR (data not shown). Therefore, their activities are not expected in asexual life stages.

**Figure 2:**
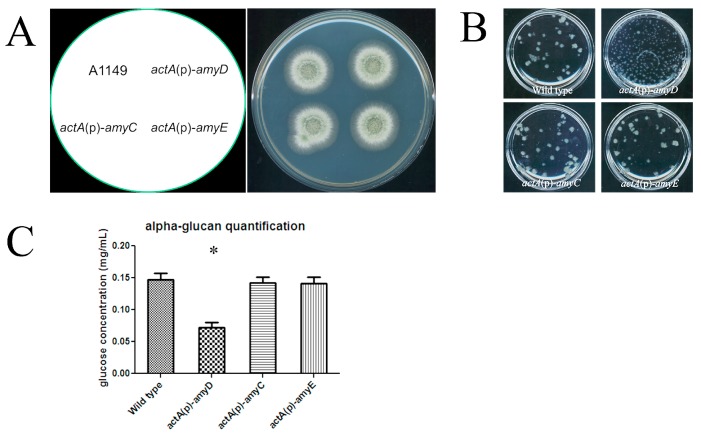
Overexpression of *amyC* and *amyE* does not affect significantly α-glucan accumulation. (**A**) 10^5^ freshly harvested conidia of each strain were inoculated on complete medium and the plates were incubated at 30 °C for 48 h. All constructed strains showed the wild type colony phenotype on solid medium; (**B**) a large number (5 × 10^7^) of freshly harvested conidia were inoculated in flask with 20 mL complete medium, then the flask was incubated at 30 °C, 150 rpm overnight. Only *actA*(p)-*amyD* formed tiny colonies; (**C**) A large number (2 × 10^7^) of spores of each strain were inoculated in 100 mL complete medium. Samples were grown in flasks at 30 °C with 150 rpm for 24 h. α-glucan was extracted from 1 mg dry cell wall, and then digested to glucose and quantified by an anthrone assay. Results represent the mean of three independent quantification tests with duplicates each time ± standard deviation. The data for each strain were compared with wild type (column 1) individually by Mann Whitney *U* test. Significant difference (*p* < 0.05) is indicated by asterisks.

We overexpressed *amyC* and *amyE* to examine their effects on α‑glucan. An actin promoter (*actA*(p)) from *A. nidulans* was used to overexpress these genes as we previously used for *amyD* [10]. The *actA*(p)-*amyC* and *actA*(p)-*amyE* strains had no obvious phenotypic change compared to the wild-type reference strain A1149, when tested on solid medium, or when grown in shaken liquid medium with respect to the colony size (Figure 2A,B). In contrast, the *actA*(p)-*amyD* strain formed many tiny colonies in shaken liquid culture (Figure 2B). This suggests that AmyC and AmyE may not have the same function as AmyD. Our qPCR results showed *amyC* and *amyE* were each overexpressed by several hundred-fold when regulated by *actA*(p), confirming their low expression under native promoters (Table 1). However, the α-glucan content in *actA*(p)-*amyC* and *actA*(p)-*amyE* was comparable to the wild-type cells (Figure 2C), unlike *actA*(p)-*amyD* (Figure 2C). So far, AmyD is the only reported amylase-like protein that has a repressive effect on α‑glucan accumulation in *A. nidulans*.

### 2.3. MutA, as Well as AgnB, but Not AgnE, Can Repress α-Glucan Accumulation

In order to verify whether AmyD could facilitate α-glucan degradation, we needed to find a functional α-glucanase. MutA (encoded by ANID7349) is the only characterized α‑glucanase in *A. nidulans.* However, its expression has only been studied for the sexual life cycle [23]. Two more α-glucanase encoding genes, *agnB* (ANID3790) and *agnE* (ANID1604), have also been annotated in the *A. nidulans* genome [22]. Therefore, AgnB and AgnE were chosen as our study candidates and MutA was included as a positive control.

**Figure 3:**
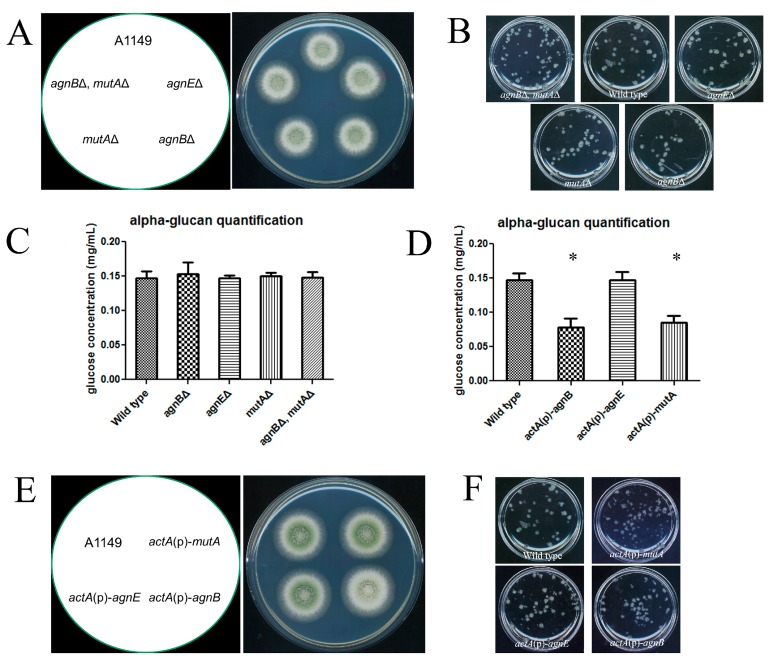
AgnB and MutA are functional α-glucanases. (**A**) Freshly harvested conidia (10^5^) of each strain were inoculated on complete medium and the plates were incubated at 30 °C for 48 h. All constructed strains showed the wild-type colony phenotype on solid medium; (**B**) freshly harvested conidia (5 × 10^7^) were inoculated in a flask with 20 mL complete medium, then the flask was incubated at 30 °C, 150 rpm overnight. All strains behaved the same as the wild-type; (**C**,**D**) spores of the indicated strain (2 × 10^7^) were inoculated in 100 mL complete medium. Samples were grown in flasks at 30 °C with 150 rpm for 24 h. α-glucan was extracted from 1 mg of dry cell wall, and then digested to glucose and quantified using an anthrone assay [24]. Results represent the mean of three independent quantification tests with duplicates each time ± standard deviation. The data for each strain were compared with the wild-type (column 1) individually by a Mann Whitney *U* test. Significant difference (*p* < 0.05) was indicated by asterisks; (**E**) conidia of each strain were prepared and inoculated as in (**A**). Only *actA*(p)-*agnB* showed pigment deficiency; and (**F**) all strains behaved the same as the wild-type in shaken liquid medium. Growth condition was the same as described in (**B**).

A time-course expression study showed that both *agnB* and *mutA* had very low expression throughout *A. nidulans* asexual life cycle (Table 1), whereas expression of *agnE* was highly induced during conidiation (Table 1). When the α-glucanases were individually deleted, this change had no impact on colony phenotypes or α-glucan content (Figure 3A–C), as expected due to their low expression levels in vegetative growth. When the α‑glucanases were individually overexpressed by *actA*(p), each had a many hundred-fold increase in expression level (Table 1), but only the overexpression of AgnB and MutA led to a lower α‑glucan content (Figure 3D). Therefore, AgnB and MutA are the functional α‑glucanases in our test. For colonies grown on solid medium, the *actA*(p)-*agnB* strain had a pale colony color (Figure 3E), even though quantification of conidiation was unchanged (Table S1), showing that the color change was not due to the conidia number. We expect that the color difference could be due to a defect in conidia pigment formation somehow related to the overexpression of *agnB*. Intriguingly, both *actA*(p)-*agnB* and *actA*(p)-*mutA* behaved the same as wild-type in shaken liquid medium (Figure 3F), unlike *actA*(p)-*amyD* that formed tiny colonies (Figure 2B). We also deleted AgnB and MutA together, just in case they might compensate for each other when individually deleted. However, the double deletion strain still had no impact on α-glucan content (Figure 3C).

### 2.4. Functions of AgnB and MutA Are Independent from AmyD

**Figure 4:**
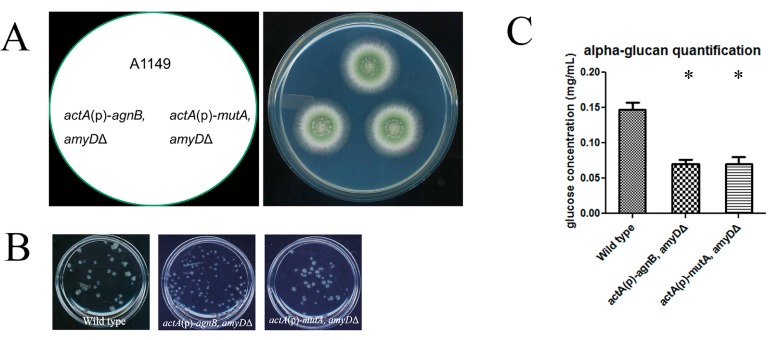
The absence of AmyD cannot suppress the increased α‑glucan production of *agnB*- or *mutA*-overexpression strains. (**A**) All constructed strains showed the wild-type colony phenotype on solid medium; (**B**) all strains behaved the same as the wild-type; and (**C**) α-glucan was extracted from 1 mg of dry cell wall, and then digested to glucose and quantified using an anthrone assay. Results represent the mean of three independent quantification tests with duplicates each time ± standard deviation. The data for each strain were compared with wild type (column 1) individually by a Mann Whitney *U* test. Significant difference (*p* < 0.05) was indicated by asterisks.

Our results showed that overexpressed AgnB, MutA, and AmyD all had similar repressive effects on α‑glucan content (Figure 2C and Figure 3D). Considering they each have a signal peptide and a GPI-anchor site [20], we hypothesized that AmyD may work together with either or both α-glucanases to degrade α-glucan [10]. If our hypothesis was correct, the repressive effects on α-glucan content from AgnB and MutA should be abolished or reduced when AmyD was deleted. To verify this, we generated (*actA*(p)-*agnB*, *amyD*Δ) and (*actA*(p)-*mutA*, *amyD*Δ) strains. We found these two strains had wild-type phenotypes on solid medium and in shaken liquid medium (Figure 4A,B). The pigment defect in *actA*(p)-*agnB* was recovered with the deletion of *amyD* (Figure 3E and Figure 4A). However, both strains still showed low α-glucan content similar to *actA*(p)-*agnB* and *actA*(p)-*mutA* (Figure 3D and Figure 4C), indicating the effects of AgnB and MutA were still present. Therefore, the functions of AgnB and MutA appear to be independent from AmyD.

### 2.5. Dynamics of α-Glucan Accumulation Affects Colony Formation in Liquid Medium and Drug Sensitivity

**Figure 5:**
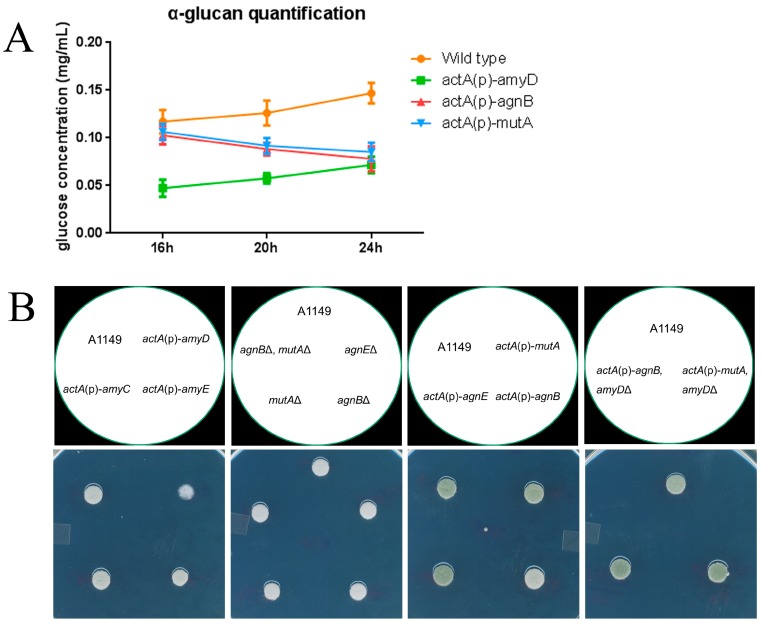
Dynamics of α-glucan accumulation and its impact on CFW sensitivity. (**A**) Dynamics of α-glucan accumulation in each strain with time. 2 × 10^7^ spores of each strain were inoculated in 100 mL complete medium. Samples were grown in flasks at 30 °C with 150 rpm for 16 h, 20 h, and 24 h, respectively. α-glucan was extracted from 1 mg of dry cell wall, and then digested to glucose and quantified by an anthrone assay. Wild-type and *actA*(p)-*amyD* showed the same increasing trend from 16 h to 24 h, except the glucose concentration in *actA*(p)-*amyD* was much lower than that of the wild-type at each time point. The *actA*(p)-*agnB* and *actA*(p)-*mutA* had the same decreasing trend from 16 h to 24 h; (**B**) freshly harvested conidia (10^5^) of each strain were inoculated on 50 μg/mL CFW plates and the plates were incubated at 30 °C for 48 h. Only *actA*(p)-*amyD* showed delayed germination and growth.

It was interesting to see that *actA*(p)-*agnB*, *actA*(p)-*mutA*, and *actA*(p)-*amyD* strains had similar α‑glucan content (Figure 2C and Figure 3D), but behaved differently in shaken liquid medium (compare Figure 2B with Figure 3F). We thought this might be because of how α‑glucan accumulated in these strains. In all of our previous quantification experiments, we collected the fungal cell samples at 24 h post-inoculation. In contrast, visualization of colonies in shaken liquid had typically been done earlier, usually around 16 h post-inoculation. Thus, we also collected additional samples at 16 h and 20 h post-inoculation to examine the dynamics of α-glucan accumulation in cell walls. Results showed very different patterns of α-glucan accumulation in these strains (Figure 5A). In wild-type, α-glucan content increased from 16 h to 24 h. In *actA*(p)-*amyD*, the trend to increasing α-glucan was maintained, but at each time point the concentration of α-glucan was only about 50% of the wild-type. However, in *actA*(p)-*agnB* and *actA*(p)-*mutA*, α-glucan content showed a decreasing trend from 16 h to 24 h. We also noticed that at 16 h the α-glucan content in wild-type, *actA*(p)-*agnB*, and *actA*(p)-*mutA* was very similar, whereas the α-glucan content in *actA*(p)-*amyD* was much lower.

In our previous work, we found that α-glucan content was correlated with sensitivity to Calcofluor White (CFW) [10]. We wondered if this change was also correlated with α-glucan accumulation dynamics. We tested all strains on 50 μg/mL CFW. Only *actA*(p)-*amyD* showed delayed germination and/or slower growth, whereas all other strains maintained the same growth ability (Figure 5B).

## 3. Discussion

In our previous study, we found AmyD repressed α-glucan accumulation in *A. nidulans* [10]. To investigate the underlying mechanism, we extended our work to other α-glucan metabolism‑related genes to further understand the function of AmyD.

### 3.1. AmyD Is the Major Negative Regulator of α-Glucan Accumulation in the A. nidulans Asexual Life Cycle

Both *amyC* and *amyE* share high sequence similarity with *amyD*, however our results showed they did not affect α-glucan accumulation. They also had no impact on starch digestion when tested on starch-only medium (unpublished data), so their functions remain unclear. So far, AmyD is the only reported amylase-like protein that can repress α-glucan accumulation in *A. nidulans*. From our results, the α-glucanases, AgnB and MutA showed similar repressive effects as AmyD when overexpressed (Figure 3D). However, each maintained a very low expression level in the *A. nidulans* asexual life cycle (Table 1), especially when compared to the expression level of *amyD* at each stage. This also explained why deletion of AgnB and MutA had no impact on α-glucan accumulation (Figure 3C), but deletion of AmyD did [10]. Altogether, we conclude AmyD is the major negative regulator of α-glucan accumulation during *A. nidulans* asexual life cycle. However, we could not rule out that AgnB and MutA may have particular roles in certain types of cells during colony development, which needs further investigation.

### 3.2. Functions of α-Glucanases and AmyD Are Independent from Each Other

Our results confirmed MutA as a functional α-glucanase and also revealed that AgnB, but not AgnE, had the same effect as AmyD to repress α-glucan accumulation. However, the low expression level of *mutA* and *agnB* in the *A. nidulans* asexual life cycle (Table 1) suggested that α-glucan degradation is not active during these stages. This is consistent with why even the *mutA* and *agnB* double-deletion strains maintained the same α-glucan content as the wild-type. This could also explain why the most prominent anti-α-glucan antibody-staining signal was from the older hyphal regions [10], because α-glucan was not recycled during the asexual life cycle. With this in mind, we think the function of AmyD is not based on α-glucanase.

On the other hand, *amyD* had a relatively high expression level during *A. nidulans* asexual development [10]. It is still possible the function of α-glucanase depends on AmyD. However, when *amyD* was deleted from *actA*(p)-*agnB* and *actA*(p)-*mutA*, the repressive effects on α-glucan from AgnB and MutA were maintained (Figure 4C). Therefore, the functions of glucanases are independent from AmyD. Evidence from other α-glucanase characterization work also showed that α-glucanase is functional by itself [14,15,16]. In addition, we noticed that, when AgnB or MutA was overexpressed in an AmyDΔ background, the effect from AmyD deletion was abolished (compare data in [10] with Figure 3D and Figure 4C). Thus, we interpret this as α-glucanases being superior in regulating α-glucan content in the cell wall.

In our study, we did find that the deletion of *amyD* in an *actA*(p)-*agnB* strain reversed the pigment formation defect (compare Figure 3E with Figure 4A), which was a specific phenotypic change in *actA*(p)-*agnB*. Otherwise, we have never found that low α-glucan content led to a pigment defect. Therefore, we think this phenotypic change does not relate to α-glucan content.

### 3.3. α-Glucan Content in Early Life Stage Is Critical for Colony Formation in Shaken Liquid as Well as Drug Sensitivity

Our dynamics study showed that the α-glucan accumulation processes in *actA*(p)-*agnB* and *actA*(p)-*mutA* were unlike *actA*(p)-*amyD* (Figure 5A). Although these colonies had similar α‑glucan content after 24 h growth, the α-glucan content at 16 h and 20 h was different. Especially at 16 h, the α-glucan content in *actA*(p)-*agnB* and *actA*(p)-*mutA* was almost the same as the wild-type, whereas in *actA*(p)-*amyD* the α-glucan level was only half that of the wild-type. This could explain why *actA*(p)-*agnB* and *actA*(p)-*mutA* formed the regular size colonies as wild type (Figure 3F), because colony formation in shaken liquid was already complete at 16 h. Even though the α-glucan content decreased in these two strains at later time, the formed colonies were unable to disassemble. The same principle also explained why *actA*(p)-*agnB* and *actA*(p)-*mutA* maintained the same drug sensitivity as the wild-type (Figure 4B). When *A. nidulans* was stressed by CFW, spore germination was delayed. However, the higher α-glucan content in the early life stage enabled the *actA*(p)-*agnB* and *actA*(p)-*mutA* strains to form colonies on solid medium faster than *actA*(p)-*amyD*.

Why the effects of AgnB and MutA started later than AmyD still needs further study, however, it is clear the mechanism of AmyD is different from α-glucanase. According to the different α-glucan accumulation patterns (Figure 5A), it is more likely that AmyD directly represses α-glucan synthesis rather than facilitating α-glucan degradation. One possible reason is that AmyD may affect the major α-glucan synthase (AgsB) expression. However, the qPCR analysis revealed no significant difference of *agsB* expression in *amyD*Δ and *actA*(p)-*amyD* strains compared to A1149 (Table S2).

AgtA, the homologue of AmyD in *A. niger*, has been enzymatically characterized [19]. These results showed AgtA had very low starch hydrolysis ability but served as a glucanotransferase on α-1,4-glucosidic linkages. The amino acid sequence identity between AmyD and AgtA is 70%, so it is highly likely AmyD will have a similar function as AgtA, but this will need enzymatic study to confirm and is beyond the scope of our current work. Further study on the mechanism of AmyD will also require establishing an in vitro α-glucan synthesis system to find out how AmyD prevents α-glucan accumulation. Nevertheless, considering the repressive effect of AmyD on α-glucan accumulation, AmyD may potentially prevent fungal infection. For instance, α-glucan was shown to be maintained at a minimal level in the conidia of *M. oryzae*, and was required during pathogenesis [12]. Therefore, effective α-glucan synthesis is important during this process and this newly-synthesized α-glucan is believed to function as a mask to protect the fungal cells from host immune recognition [11,12]. If the host cell could express AmyD to suppress α-glucan synthesis, the infection may fail due to the detection of host immune system, as Fujikawa and colleagues showed [12].

In summary, AmyD is the only reported amylase-like protein that can repress α‑glucan accumulation in *A. nidulans*, and it is also the major negative regulator during the asexual life cycle. The dynamics study showed the effect of AmyD started earlier than α-glucanase, and the mechanism of AmyD was different and independent from α-glucanase. These data suggested AmyD may not serve for α-glucan degradation, but instead may directly repress α‑glucan synthesis at the protein level.

## 4. Materials and Methods

### 4.1. Strains, Plasmids, and Medium

All strains in this study were constructed in *A. nidulans* A1149. The A1149 strain was also the wild-type control for all assays in this paper. Strains used in this study are listed in Table S3. Primers and plasmids are listed in Table S4. Strategies for gene deletion and confirmation methods were described by Szewczyk et al. [25] and El-Ganiny et al. [26]. Briefly, a targeted replacement construct was constructed by fusion PCR including 1 kb upstream, a selectable marker, and 1 kb downstream (details see Figure S2A). This construct was transformed to A1149 protoplasts. *A. fumigatus* *pyrG* and *pyroA* were used as selectable markers (details for each strain see Figure S2B). The strategy for promoter exchange was previously described in [10]. For promoter exchange, the transformation construct was 1 kb upstream of the target, the selectable marker, *actA*(p) and 1 kb of the target gene from 5′ end (Figure S2A). The *actA* promoter was amplified from A1149 genomic DNA and the sequence was given in Figure S3. Again, details for each construction see Figure S2B. PCR confirmation of each constructed strain is shown in Figure S4.The sequence of each overexpression strain was confirmed by DNA sequencing, and only a clone lacking mutations was used for further study.

For generation of a strain expressing an chimera-AmyD-GFP strain, a construct containing all following elements: 1 kb upstream of *amyD*, *AfpyrG*, *actA*(p), *amyD* signal peptide sequence, *gfp* (no stop codon) and *amyD* GPI-anchor site sequence, and 1 kb downstream of *amyD*, was generated and transformed to A1149 protoplasts. For details of strain construction and verification see Figure 2A,B.

All strains were grown on complete medium (CM: 1% glucose, 0.2% peptone, 0.1% yeast extract, 0.1% casamino acids, 50 mL 20× nitrate salts, 1 mL trace elements, 1 mL vitamin solution, pH 6.5) or minimal medium (MM: 1% glucose, 50 mL 20× nitrate salts, 1 mL trace elements, 0.001% thiamine, pH 6.5) supplemented with nutrition solution as required. Trace elements (2.2 g ZnSO_4_·7H_2_O, 1.1 g H_3_BO_3_, 0.5 g MnCl_2_·4H_2_O, 0.5 g FeSO_4_·7H_2_O, 0.17 g CoCl_2_·6H_2_O, 0.16 g CuSO_4_·5H_2_O, 0.15 g Na_2_MoO_4_·2H_2_O, and 5 g Na_4_EDTA in 100 mL water, pH adjusted to 6.5 by KOH pellets), vitamin solution (100 mg each of biotin, pyridoxin, thiamine, riboflavin, PABA (*p*-aminobenzoic acid), and nicotinic acid per 100 mL water), nitrate salt, and all nutrition stocks are described in Kaminskyj [27]. For transformation medium, 1 M sucrose was added to MM as osmoticum. All strains were grown at 30 °C, unless otherwise mentioned.

### 4.2. Quantification of Conidiation

Molten CM agar (1.5 mL) was added to each well in a 24-well plate and seeded with 10^5^ conidia after solidification. Plates were incubated for 4 d, then 1 mL ultra-pure water from Barnstead™ Nanopure™ system was used to collect conidia from each well. Conidia were quantified by hemocytometer.

### 4.3. α-Glucan Quantification

The method was adapted from Momany et al. [28] and Marion et al. [29], as described in [10]. Briefly, 2 × 10^7^ conidia were grown at 30 °C in 100 mL liquid CM, shaken at 150 rpm for 24 h (or the indicated time). Colonies were collected by filtration and washed with 0.5 M NaCl. Cells were frozen at −80 °C for 2–4 h, then broken in disruption buffer (DB: 20 mM Tris, 50 mM EDTA, pH 8.0) using a Virsonic Ultrasonic Cell Disrupter, until hyphal ghosts formed. Cell walls were separated by centrifugation at 3500× *g* for 10 min. The pellet containing the cell wall fraction was washed in DB with stirring for 4 h at 4 °C followed by a wash with sterile ultrapure water under the same conditions, pelleted again, and lyophilized. Dry cell wall samples were weighed, then suspended in 1 M NaOH at 0.5 mg·mL^−1^. Alkaline extraction was performed overnight at 37 °C. Then 2 mL of alkaline-soluble fraction (containing 1 mg cell wall) was used for the following process. The alkali was neutralized by acetic acid until pH 5.5. α-Glucan was collected by centrifugation at 12,000× *g* for 10 min, and then washed twice in ultrapure water. Finally, α-glucan was hydrolyzed by 2 mL 3 M H_2_SO_4_, at 100 °C for 1 h. Glucose content (mainly from α-glucan in the alkali-soluble fraction) was quantified using the anthrone assay [30]. Briefly, 100 μL of α-glucan hydrolysis solution was added to 1 mL of anthrone solution (2 mg/mL in concentrated H_2_SO_4_). Then the mixture was boiled in water for 10 min with immediate cool on ice. OD630 was measured for each sample. All experiments were repeated three times with duplicates each time.

### 4.4. RT-PCR and qPCR

For the time-course expression study, 2 × 10^7^ conidia were inoculated in liquid CM and incubated at 30 °C with or without shaking at 150 rpm. Colonies were collected at 14 h and 24 h for each group. For the static incubation, only the colonies on the liquid surface were collected. Colonies were immediately frozen in liquid nitrogen, and lyophilized.

For the overexpression study, 2 × 10^7^ conidia were inoculated in liquid CM and incubated at 30 °C with shaking at 150 rpm for 14 h. Colonies were collected by filtration, immediately frozen in liquid nitrogen, then lyophilized.

Total RNA was extracted using an RNeasy plant kit (Qiagen, Hilden, Germany) following the manufacturer’s instructions. RNA concentration was measured using a Nanodrop^®^ (Wilmington, DE, USA), then diluted to 500 ng·μL^−1^. Genomic DNA elimination and reverse transcription used a QuaniTect reverse transcription kit (Qiagen) following the manufacturer instructions.

Quantitative real time PCR (qPCR) was performed in 96-well optical plates in an iQ5 real‑time PCR detection system (Bio-Rad, Hercules, CA, USA). Gene expression was assayed in total volume of 20 μL per reaction containing cDNA at an appropriate dilution and SYBR green fluorescein (Qiagen). A no-template control was used for each pair of primers. Histone was used as a reference gene [24]. Primers for qPCR are listed in Table S4.

The qPCR amplification used the following conditions: 95 °C/15 min for one cycle, 95 °C/15 s, 55 °C/40 s and 72 °C/30 s for 40 cycles and final extension cycle of 72 °C/2 min. Melting curve analysis was done as follows: 15 s at 65 °C with an increase of 0.5 °C each cycle to 95 °C. The relative expression was normalized to histone and calculated using the ΔΔ*C*_t_ method [31]. Three independent experiments with triplicates were performed for each reaction.

### 4.5. Drug Sensitivity Test

Calcofluor White (American Cyanamid Company, West Paterson, NJ, USA) was prepared as a stock at 10 mg·mL^−1^ in 25 mM KOH [32]. The stock solution was sterilized by filtration. For testing, CFW stock solution was added to CM agar cooled to 55–60 °C. Then, 10^5^ conidia of each strain were inoculated on the plate after solidification. Plates were incubated for 48 h at 30 °C.

### 4.6. Confocal Imaging

For GFP signal imaging, conidia were grown on dialysis tubing at 30 °C for 16 h. Samples were examined using a Zeiss META501 confocal epifluorescence microscope at 63 N.A. 1.2 × or 25 × N.A. 1.0 objective lens. Confocal imaging used 488 nm excitation with emission controlled by a BP 505–530 nm filter.

### 4.7. Statistical Analysis

All α-glucan quantification analyses were performed in three independent tests with duplicates each time. Histograms were created by Graphpad Prism 6 (GraphPad Software, La Jolla, CA, USA). Statistical analysis used a Mann Whitney *U* test.

## 5. Conclusions

Data from this study showed AmyD is a major repressive effector for α-glucan accumulation. The working mechanism of AmyD is totally different from α-glucanase. Therefore, AmyD should directly suppress α-glucan synthesis.

## Figures and Tables

**Table 1:** Time-course expression study.

	Shaken Growth		Static Growth		Overexpression by *actA*(p)
genes	14 h	24 h	14 h	24 h	14 h
*amyC*	1	1.44 ± 0.44	1.28 ± 0.28	18.84 ± 6.36	794.13 ± 190.28
*amyE*	1	1.72 ± 0.71	1.93 ± 0.49	27.82 ± 9.79	484.82 ± 140.61
*agnB*	1	2.16 ± 0.63	2.39 ± 0.83	11.71 ± 3.93	871.00 ± 191.59
*agnE*	1	1.92 ± 0.57	1.59 ± 0.53	274.90 ± 85.55	1268.84 ± 292.38
*mutA*	1	2.97 ± 0.89	2.31 ± 0.96	6.96 ± 1.99	1753.59 ± 654.97

## Supplementary Materials

Supplementary materials can be found at www.mdpi.com/1422-0067/18/4/695/s1.
