# Supplementary material for: An Amylase-Like Protein, AmyD, Is the Major Negative Regulator for α-Glucan Synthesis in Aspergillus nidulans during the Asexual Life Cycle
Source: Int J Mol Sci. 2017 Mar 27;18(4):695. doi: 10.3390/ijms18040695 (PMC5412281; doi:10.3390/ijms18040695)
Supplement: Supplementary file 1 [file ijms-18-00695-s001.pdf]

Table S1 Quantification of conidiation

**Medium**	**CM**	
**Strains**	Wild type	*actA*(p)-*agnB*
**Conidiation**	100% ± 9.7%	94.7% ± 5.9%

10^5 conidia were grown on 1.5mL medium in 24-well plate at 30 °C for 4 d. 1 mL ultra-pure H_2O was used to collect conidia from each well. Conidia were quantified by hemocytometer. Results represent the average of three independent quantification tests with triplicates each time ± standard deviation.

Table S2 Expression of *agsB* in *amyD*△ and *actA*(p)-*amyD* strains

	**A1149**	***amyD*△**	***actA*(p)-*amy*D**
agsB	1	1.12± 0.27	1.08± 0.15

A large number of conidia (2×10^7) were inoculated in liquid CM and incubated at 30 °C for 14 h. The expression of *agsB* in A1149 strain was defined as 1. Results present the mean of three independent qPCR tests with triplicates each time ± standard deviation.

Table S3 *Aspergillus nidulans* strains in this study

**Strains**	**alias**	**Genotype**	**Origin**
A1149	A1149	*pyrG89*; *pyroA4*; *nkuA*::*argB*	FGSC*
actA(p)-*amyD*	AXH38	*amyD*p:: *AfpyroA*:*actA*p:*amyD*; *pyrG89*; *pyroA4*; *nkuA*::*argB*	He et al. [10]
actA(p)-*amyC*	AXH52	*amyC*p:: *AfpyroA*:*actA*p:*amyC*; *pyrG89*; *pyroA4*; *nkuA*::*argB*	this study
actA(p)-*amyE*	AXH53	*amyE*:: *AfpyroA*:*actA*p:*amyE*; *pyrG89*; *pyroA4*; *nkuA*::*argB*	this study
*agnB*Δ	AXH78	*AN3790*:: *AfpyroA*; *pyrG89*; *pyroA4*; *nkuA*::*argB*	this study
*agnE*Δ	AXH42	*AN1604*:: *AfpyroA*; *pyrG89*; *pyroA4*; *nkuA*::*argB*	this study
*mutA*Δ	AXH79	*AN7349*::*AfpyrG*; *pyrG89*; *pyroA4*; *nkuA*::*argB*	this study
*agnB*Δ, *mutA*Δ	AXH80	*AN3790*:: *AfpyroA*; *AN7349*::*AfpyrG*; *pyrG89*; *pyroA4*; *nkuA*::*argB*	this study
actA(p)-*agnB*	AXH50	*agnB*p:: *AfpyroA*:*actA*p:*agnB*; *pyrG89*; *pyroA4*; *nkuA*::*argB*	this study
actA(p)-*agnE*	AXH47	*agnE*p:: *AfpyroA*:*actA*p:*agnE*; *pyrG89*; *pyroA4*; *nkuA*::*argB*	this study
actA(p)-*mutA*	AXH51	*mutA*p:: *AfpyroA*:*actA*p:*mutA*; *pyrG89*; *pyroA4*; *nkuA*::*argB*	this study
actA(p)-*agnB, amyD*Δ	AXH55	*agnB*p:: *AfpyroA*:*actA*p:*agnB*; *AN3308*::*AfpyrG*; *pyrG89*; *pyroA4*; *nkuA*::*argB*	this study
actA(p)-*mutA, amyD*Δ	AXH56	*mutA*p:: *AfpyroA*:*actA*p:*mutA*; *AN3308*::*AfpyrG*; *pyrG89*; *pyroA4*; *nkuA*::*argB*	this study
amyD-gfp	AXH86	*amyD:: AfpyroA:actAp:gfp; pyrG89; pyroA4; nkuA::argB*	this study

* Fungal Genetics Stock Center

Table S4 Primers and plasmids in this study

Primers	Sequence 5′ to 3′	Description
Selective marker and strain confirmation		
AME1	ATGTCGTCCAAGTCGCAATT	Af*pyrG**
AME2	TCATGACTATGCCGCATACTAC	Af*pyrG**
SE231	GGACATCAGATGCTGGATTACTAAAG	Af*pyroA* with native promoter
SE232	TTACCATCCTCTCTTGGCCA	Af*pyroA* with native promoter
AME7	CAATACCGTCCAGAAGCAATAC	Af*pyrG* confirmation*
AME8	CACATCCGACTAGCACTATCC	Af*pyrG* confirmation*
AME15	ATTCCTGTCATGGCCAAAG	Af*pyroA* confirmation*
AME16	TCAACAACATCTCCGGTACC	Af*pyroA* confirmation*
Gene deletion		
SE103	CGGCCATTGACCATGAAC	*amyD* upstream F
SE104	AATTGCGACTATGGACGACATTGTGACGATGTCTGGACCG	*amyD* upstream R (pyrG tail)
SE105	GAGTATGCGGCAAGTCATGATTTGATCTGTTTTCATCTTTTTGC	*amyD* downstream F (pyrG tail)
SE106	GTCATAGATGTCATACCCGTTTCC	*amyD* downstream R
SE107	GTCTTCATCCGGTCCACTATC	*amyD* Fusion F
SE108	GAAGATGAGGGTGTTGTCG	*amyD* Fusion R
SE343	GGGGAGTCGAGTTTACACCA	*agnB* upstream F
SE344	CTTAGTAATCCAGCATCTGATGTCCGATCTGGACCGTCAGTTTCG	*agnB* upstream R (pyroA tail)
SE408	TGGCCAAGAGAGGATGGTAATTAGCGCATTGTTTCTGCAG	*agnB* downstream F (pyroA tail)
SE409	GTAGAGACCGCGCTCTGTCT	*agnB* downstream R
SE347	TTCTCAGTAACCCCCAAGACG	*agnB*Δ Fusion F
SE410	CGAAGACTGACTTTGGTACCG	*agnB*Δ Fusion R
SE320	ATGCCATTGAGCTGGACATT	*agnE* F
SE321	TCATATCAGGCAAGAGAGCAGG	*agnE* R
SE322	CTGGCGAGAGATTCTGGAAC	*agnE* upstream F
SE323	TCCATTACCCATTTCGAAGC	*agnE* upstream R (pyroA tail)
SE324	TGGCCAAGAGAGGATGGTAACACCTAATACCAGGCCAGTTTT	*agnE* downstream F (pyroA tail)
SE325	CCTCATATAGAGACCGCGCA	*agnE* downstream R
SE326	CTAGCCTAGCATCTTTACCGACTG	*agnE*Δ Fusion F
SE327	GTAGAGTTGGGACTTAAGCTAGTCG	*agnE*Δ Fusion R
SE349	AGTAATTTCGCGCGATACCC	*mutA* upstream F
SE411	GGGCGAGCCTTTAACGTACAGTTGCTTGCTTGAGGCT	*mutA* upstream R (pyrG tail)
SE190	AATTGCGACTTGGACGACATGGTGTTTAGGGGTG	*actA*p R (pyrG tail)
SE191	TACGTTAAAGGCTCGCCC	*actA*p F
SE412	GAGTATGCGGCAAGTCATGATGCTAGAAGGATCGAGCCA	*mutA* downstream F (pyrG tail)
SE413	GTTTTCCTCTGACCCAGTCG	*mutA* downstream R
SE353	GTTCGAGTTGGTTGCGAGTC	*mutA*Δ Fusion F
SE414	CCAAGTTGAGTCTTACGCCG	*mutA*Δ Fusion R
Promoter exchange		
SE234	TGGCCAAGAGAGGATGGTAATACGTTAAAGGCTCGCCC	*actA*(p) F (pyroA tail)
SE208	GGTGTTTAGGGGTGGATTAGAA	*actA*(p) R
SE355	AGAGCTCATCGTGAAGGATGA	*amyC* upstream F
SE356	CTTAGTAATCCAGCATCTGATGTCCGGTTTGATAGACGGATCTTGTCTT	*amyC* upstream R (pyroA tail)
SE357	TTCTAATCCACCCCTAAACACCATGACTGACAGATTCGCCC	*amyC* F (*actA*(p) tail)
SE358	CGTGTTGTAAAGTGGGGAGG	*amyC*_1100 R
SE359	CCTGGAAGTACCTAGGAAACTGG	*actA*(p)-*amyC* Fusion F
SE360	AAATTATTGAGTCTAATGGGCGAC	*actA*(p)-*amyC* Fusion R

SE361	GGCGCTCCAGTTATACCG	*amyE* upstream F
SE362	CTTAGTAATCCAGCATCTGATGTCCCCTTAGAAAGGTAGGTTGCTGTG	*amyE* upstream R (pyroA tail)
SE363	TTCTAATCCACCCCTAAACACCATGCGGCGCCTCACATGT	*amyE* F (*actA*(p) tail)
SE364	GTCAGTAGAAGTATGCAGCAGGTTCT	*amyE*_1100 R
SE365	AAATACCGTTCACCTTGGACG	*actA*(p)-*amyE* Fusion F
SE366	CATGGGGTAATTCAGGAGACC	*actA*(p)-*amyE* Fusion R
SE343	GGGGAGTCGAGTTTACACCA	*agnB* upstream F
SE344	CTTAGTAATCCAGCATCTGATGTCCGATCTGGACCGTCAGTTTCG	*agnB* upstream R (pyroA tail)
SE345	TTCTAATCCACCCCTAAACACCATGTATCTGAAAACGCTCTTTTTG	*agnB* F (*actA*(p) tail)
SE346	TTCTCGTCTTGAATGTACTGGTC	*agnB*_1140 R
SE347	TTCTCAGTAACCCCCAAGACG	*actA*(p)-*agnB* Fusion F
SE348	GAATGCTGCTCACATGTCCA	*actA*(p)-*agnB* Fusion R
SE322	CCGACTGACCATTTGCATC	*agnE* upstream F
SE323	CTTAGTAATCCAGCATCTGATGTCCTTTGCTTCAGGTTCGCTTC	*agnE* upstream R (pyroA tail)
SE340	TTCTAATCCACCCCTAAACACCATGCCATTGAGCTGGACATT	*agnE* F (*actA*(p) tail)
SE341	ACAGCTTGATGAGAGCTCTTGC	*agnE*_1100 R
SE326	CTAGCCTAGCATCTTTACCGACTG	*actA*(p)-*agnE* Fusion F
SE342	TCACAGGGCCGATATAATGG	*actA*(p)-*agnE* Fusion R
SE349	AGTAATTTCGCGCGATACCC	*mutA* upstream F
SE350	CTTAGTAATCCAGCATCTGATGTCCCAGTTGCTTGCTTGAGGCT	*mutA* upstream R (pyroA tail)
SE351	TTCTAATCCACCCCTAAACACCATGAAGATCTTCCACCGCTG	*mutA* F (*actA*(p) tail)
SE352	CTAGGCGCTAAAAGAGCCAA	*mutA*_1100 R
SE353	GTTCGAGTTGGTTGCGAGTC	*actA*(p)-*mutA* Fusion F
SE354	TACGTCAACCGAAAACTCCAG	*actA*(p)-*mutA* Fusion R
SE429	CCAGTGAAAAGTTCTTCTCCTTTACTGCAGTATAGACCAGCGGTCG	amyD_186R gfp tail
SE427	CATGGCATGGATGAACTATACAAAGCTGGGGGGAACGTTAGT	amyD_1633F gfp tail
SE328	AGTAAAGGAGAAGAACTTTTCACTGG	gfp F no start codon
SE315	TTTGTATAGTTCATCCATGCCATG	gfp R no stop codon
qPCR		
SE244	CACCCGGACACTAGGTATCTC	Histone qPCR F#
SE245	GAATACTATCGTAACGGCCTTGG	Histone qPCR R#
SE153	GGATGGAGATGACCCTGCTA	*amyD* qPCR F
SE154	TGCGCATCATGGTAGTCATT	*amyD* qPCR R
SE334	GGATTCCAGCCAAGTGTTGT	*amyC* qPCR F
SE335	AAAGCCCACTCCCTCTCATT	*amyC* qPCR R
SE336	TCTGGGTAAAGGGACTGGTG	*amyE* qPCR F
SE337	GTAGACTTCCCCCATCGTGA	*amyE* qPCR R
SE332	CTGGCGAGAGATTCTGGAAC	*agnB* qPCR F
SE333	TCCATTACCCATTTCGAAGC	*agnB* qPCR R
SE330	CATGATGGGTGGAGGAGTCT	*agnE* qPCR F
SE331	CAGGAGAGAGCCGATACCAG	*agnE* qPCR R
SE338	CCAAATGGAATCAACCTGCT	*mutA* qPCR F
SE339	ATGGGGAAGCTGTTTGTCAC	*mutA* qPCR R

#, Fujioka et al. (2007); *, Alam et al. (2012)

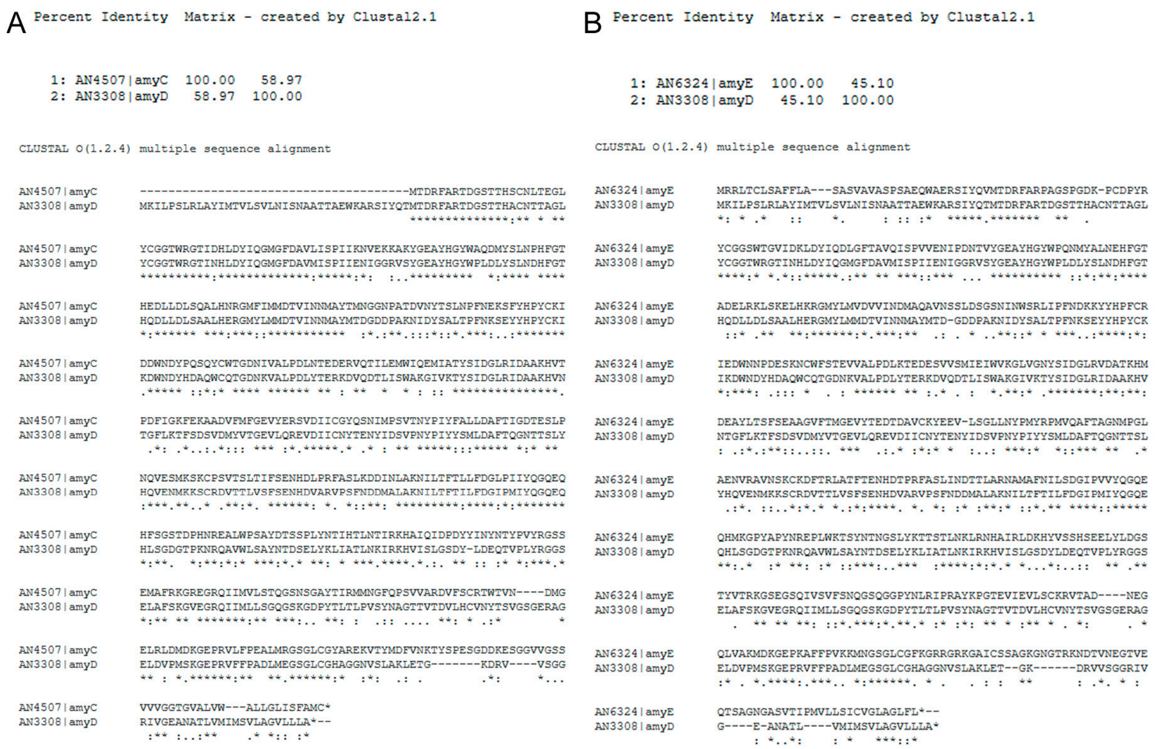

Figure S1 Amino acid sequence alignment of AmyD with AmyC and AmyE using Clustal Omega online server

A

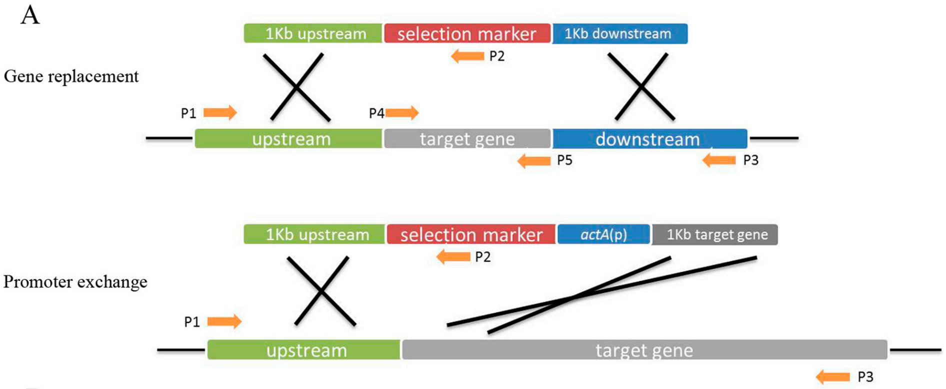

B

actA(p)-amyC:
1Kb amyC upstream + AfpyroA + actAp+ 1Kb amyC
(SE355+SE356) (SE231+SE232) (SE234+SE208) (SE357+SE358) Fusion PCR: SE359+SE360
actA(p)-amyE:
1Kb amyE upstream + AfpyroA + actAp+ 1Kb amyE
(SE361+SE362) (SE231+SE232) (SE234+SE208) (SE363+SE364) Fusion PCR: SE365+SE366
agnBΔ:
1 Kb agnB upstream + AfpyroA + 1Kb agnB downstream
(SE343+SE344) (SE231+SE232) (SE408+SE409) Fusion PCR: SE347+SE410
agnEΔ:
1 Kb agnE upstream + AfpyroA + 1Kb agnE downstream
(SE322+SE323) (SE231+SE232) (SE324+SE325) Fusion PCR: SE326+SE327
mutAΔ:
1Kb mutA upstream + actAp+ AfpyrG + 1Kb mutA downstream
(SE349+SE411) (SE190+SE191) (AME1+AME2) (SE412+SE413) Fusion PCR: SE353+SE414
agnBΔ, mutAΔ:
1 Kb agnE upstream + AfpyroA + 1Kb agnE downstream
(SE322+SE323) (SE231+SE232) (SE324+SE325) Fusion PCR: SE326+SE327
1Kb mutA upstream + actAp+ AfpyrG + 1Kb mutA downstream
(SE349+SE411) (SE190+SE191) (AME1+AME2) (SE412+SE413) Fusion PCR: SE353+SE414
actA(p)-agnB:
1Kb agnB upstream + AfpyroA + actAp+ 1Kb agnB
(SE343+SE344) (SE231+SE232) (SE234+SE208) (SE345+SE346) Fusion PCR: SE347+SE348
actA(p)-agnE:
1Kb agnE upstream + AfpyroA + actAp+ 1Kb agnE
(SE322+SE323) (SE231+SE232) (SE234+SE208) (SE340+SE341) Fusion PCR: SE326+SE342
actA(p)-mutA:
1Kb mutA upstream + AfpyroA + actAp+ 1Kb mutA
(SE349+SE350) (SE231+SE232) (SE234+SE208) (SE351+SE352) Fusion PCR: SE353+SE354
actA(p)-agnB, amyDΔ:
1Kb agnB upstream + AfpyroA + actAp+ 1Kb agnB
(SE343+SE344) (SE231+SE232) (SE234+SE208) (SE345+SE346) Fusion PCR: SE347+SE348
1 Kb amyD upstream + AfpyrG + 1Kb amyD downstream
(SE103+SE104) (AME1+AME2) (SE105+SE106) Fusion PCR: SE107+SE108
actA(p)-mutA, amyDΔ:
1Kb mutA upstream + AfpyroA + actAp+ 1Kb mutA
(SE349+SE350) (SE231+SE232) (SE234+SE208) (SE351+SE352) Fusion PCR: SE353+SE354
1 Kb amyD upstream + AfpyrG + 1Kb amyD downstream
(SE103+SE104) (AME1+AME2) (SE105+SE106) Fusion PCR: SE107+SE108

Figure S2 Gene replacement and promoter exchange schema and PCR constructs of each strain. (A) Design of construct was shown in the figure. Positions of verification PCR primers were also listed. Generally, P1+P2 were used to confirm the construct insertion into the genome. Wildtype strain results no band and the constructed strains usually result a 2Kb band. P1+P3 were used to detect whether ectopic insertion was occurred in the same genome. Because the sequence length of the selection marker (or plus *actA* promoter) is mostly different from that of the target sequence, this pair of primer should result different bands in different strains. In addition, if ectopic insertion happened, the result of the constructed strain should also show a band as that of wildtype. Occasionally, P4+P5 were used to confirm the absence of the target gene, which was only used in gene replacement construct. (B) Details of primers used for construct generation were listed.

>AN6542 upstream COORDS:ChrI_A_nidulans_FGSC_A4
TACGTTAAAGGCTCGCCCCAACCTCGAAAAGGCGGTAAAATACTAAAAACGGTCCTTAGA
AGGCCTTTTCGACAACGATTTAGAAGGGCTGAGTACAAAAGGAGATCAACCACAGATAGT
ATCGAGCGTGGTTATAGAGAACTAGTTCAGCACATGCCTTATTCCTTTTTTTTCTTCCCATTT
TTTTTTTCTTTCTTAAAGGACAGATATAAAAATCTGGAAGCCTGGAGTAGGATCCCCTTAG
GCATTGTTGAACTTCAGGAAAAAGTGAGTCGTCATCGTGCTTACAAGCAGAGAGCGATAA
TAATAGAACGAATGAGAGGACAGACCCTGTTCTTTGAAAACTGGACACGCTGGGCTGAA
CCATCATTACGGCCTTGGTCGTCGGTCTCTGCCCCACGAGGATTCATCATCTCGCGAGGCC
ACCTCAGCGCGCAATCATTCTGCCTGAGAGGCAACACTTGGTCGCCAGTATAAAAGAACC
AATTGAACAAGAGACACCTATCTCGTAGCAGTCTTTGTGTAGTCTGTACTTTATTCGTTTTA
CCCTCTGAGGAAACGCGGTGGCGGTGACCATTCGACTAGACGAGACTAAGCCCCTTTGG
CGCCTGAACCTCCAGCCCCTTTCCAGTCCTTCTGTTCAGTTCGAGCGGCTGTCGAGCTGCT
GCTGACTACTCCGCCTACCGCTACAACCTCCACCACCACCGACCACCAACAAACCCTCGAC
TCTCTCCCCTTCTCTCCTCCACTTCTCAACATCCAACTCCCCATTCTCGCTCTGTTCATCATCT
CTCCTCCTCCCTTCCTTACCTGTCAACCTCTCATTTCTTTTTCTCTTTGTTCTTCGTAGTTCGA
TTCTAATCCACCCCTAAACACC

Figure S3 Sequence of *actA* upstream

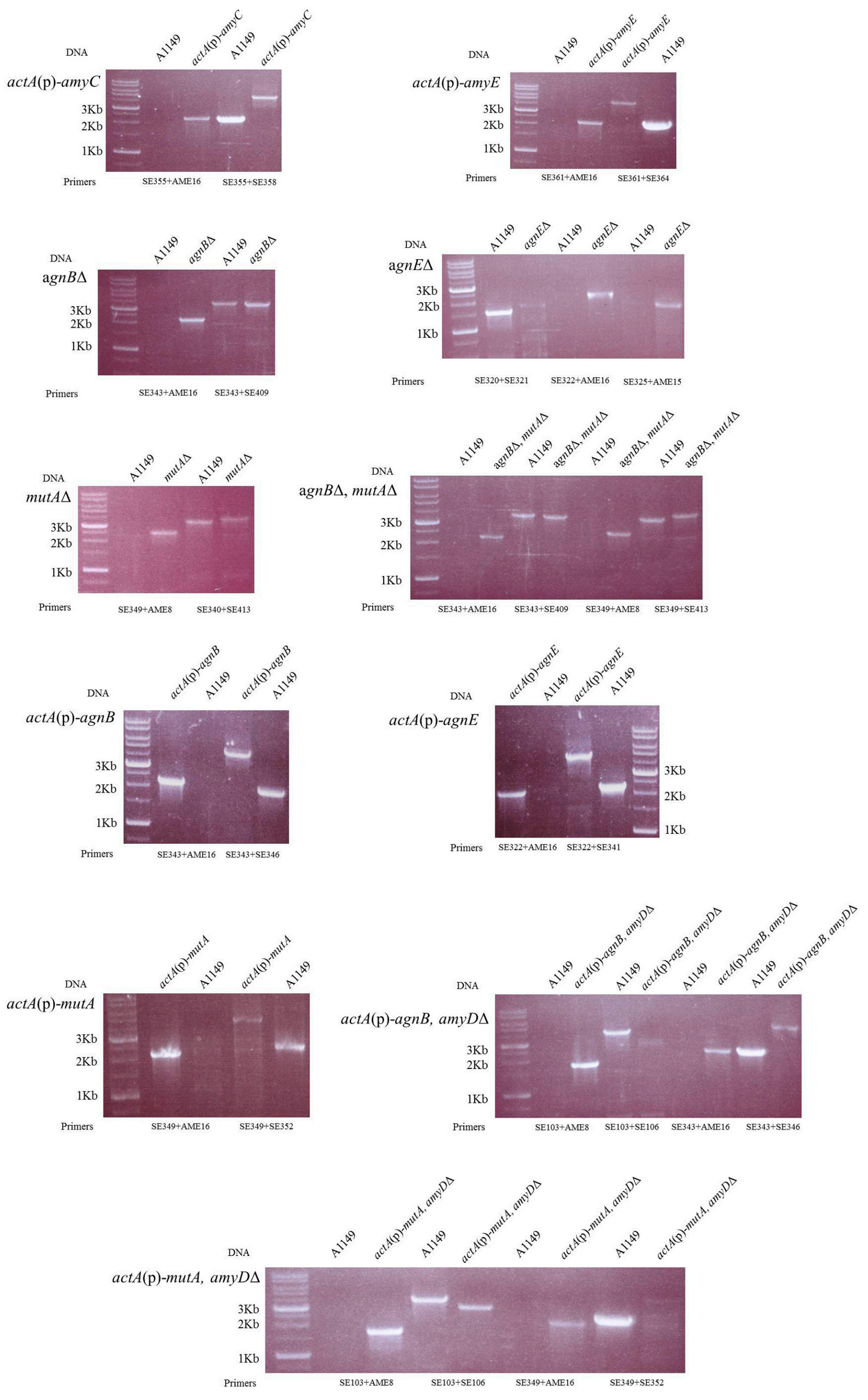

Figure S4 PCR confirmation of all constructed strains. Strains labeled at right of each gel picture. Primers are indicated at the bottom and DNA templates are on the top.
